# From trauma to brain: *Neoscytalidium dimidiatum* as an emerging cause of cerebral phaeohyphomycosis: Case report and comprehensive literature review

**DOI:** 10.1016/j.idcr.2026.e02696

**Published:** 2026-07-24

**Authors:** Hamid Eshaghi, Shahram Mahmoudi, Mahmoud Khansari, Kimia Kamali Sarvestani, Fuad Haghighat, Hasti Kamali Sarvestani

**Affiliations:** a Department of Infectious Diseases, Pediatrics Center of Excellence, Children's Medical Center, Tehran University of Medical Sciences, Tehran, Iran; b Department of Parasitology and Mycology, School of MedicineSchool of Medicine, Iran University of Medical Sciences, Tehran, Iran; c Department of Hand and Reconstructive Surgery, Sina Hospital, Tehran University of Medical Sciences, Tehran, Iran; d Department of Molecular and Cellular Biology, Zand Institute of Higher Education, Shiraz, Iran; e Department of Medical Parasitology and Mycology, School of Public Health, Tehran University of Medical Sciences, Tehran, Iran

**Keywords:** Neoscytalidium dimidiatum, Pediatric CNS infection, Cerebral phaeohyphomycosis, Trauma, Shunt, Antifungal therapy

## Abstract

**Background:**

Neoscytalidium dimidiatum is a dematiaceous fungus typically causing superficial dermatomycosis. Invasive central nervous system (CNS) infections are rare, usually fatal, and primarily reported in immunocompromised patients. Pediatric CNS involvement is exceptionally uncommon.

**Case presentation:**

We report a 16-month-old previously healthy boy from Iran with a history of traumatic brain injury and cerebrospinal fluid shunt placement. Ten months post-injury, he presented with vomiting and swelling along the shunt tract. CSF analysis showed elevated protein and low glucose, and microscopy revealed dematiaceous septate hyphae with arthroconidia. Culture and ITS sequencing confirmed *N. dimidiatum*. Initial therapy with amphotericin B, caspofungin, and fluconazole was ineffective. Susceptibility-guided therapy, including intraventricular amphotericin B and systemic posaconazole followed by itraconazole, led to full clinical and radiological recovery.

**Results:**

The isolate showed low MIC to amphotericin B (0.25 μg/mL). Molecular confirmation via ITS sequencing was deposited in GenBank.

**Conclusion:**

This case illustrates that *N. dimidiatum* can cause CNS infection in immunocompetent pediatric patients, particularly following traumatic inoculation or shunt placement. Early recognition, precise identification, and susceptibility-directed antifungal therapy, including intraventricular administration when indicated, are crucial for successful management of this otherwise highly fatal infection.

## Introduction

*Neoscytalidium* species are dematiaceous fungi primarily recognized as phytopathogens and environmental saprophytes in tropical and subtropical regions. In humans, they are most commonly associated with superficial infections of the skin and nails, often mimicking dermatophytosis. However, in rare circumstances, these organisms can cause deep or invasive disease, including involvement of the central nervous system (CNS). *Neoscytalidium dimidiatum* (formerly known as *Scytalidium dimidiatum* and *Nattrassia mangiferae*) is currently the accepted taxonomic designation according to contemporary fungal nomenclature. Throughout the remainder of this manuscript, the organism is referred to as *Neoscytalidium dimidiatum* [1].

*Neoscytalidium dimidiatum* has emerged as the principal pathogenic species within the genus. Initially described in 1933 as a plant pathogen, the organism was not recognized as a human pathogen until several decades later, when it was isolated from cases of dermatomycosis [2], [3]. Among approximately 15 saprophytic species currently assigned to this genus, only *N. dimidiatum* and *Scytalidium hyalinum* are considered clinically relevant in human disease [3]. Both species are commonly isolated from soil, decaying vegetation, and woody plants in warm climates, reflecting their environmental origin [4].

The taxonomic history of *N. dimidiatum* is complex and reflects its morphological diversity. The arthroconidial form was originally described by Nattrass in 1933 as *Torula dimidiata* [2]. Its role as a human pathogen was first established in 1971, when Gentles and Evans reported its isolation from a case of dermatomycosis [5]. Subsequently, Campbell and Mulder described infections caused by a related species, *Scytalidium hyalinum*, expanding the spectrum of human disease attributed to this group of fungi [6].

Although most reported cases involve superficial dermatomycosis, *N. dimidiatum* has occasionally been implicated in subcutaneous and invasive infections. Documented manifestations include mycetoma, deep soft-tissue infection, fungemia, and cerebral abscess or meningitis, predominantly in immunocompromised hosts [7]. CNS involvement is exceedingly rare and is typically associated with underlying immunosuppression, traumatic inoculation, or contiguous spread from adjacent structures.

Accurate identification of *N. dimidiatum* remains challenging because of its morphological similarity to other dematiaceous or saprophytic fungi. Microscopically, the organism produces dark, thick-walled arthroconidia in young cultures, while mature colonies may form pycnidial structures that release unicellular conidia. This morphological variability contributed to its complex taxonomic history and frequent misidentification in clinical laboratories [2]. Although phenotypic methods remain important for preliminary recognition, definitive species-level identification increasingly relies on molecular techniques. Sequencing of the internal transcribed spacer (ITS) region of ribosomal DNA is considered the reference method for accurate identification, particularly in cases of invasive or unusual infection [8].

Here, we report cerebral phaeohyphomycosis caused by *Neoscytalidium dimidiatum* in an immunocompetent 16-month-old boy who developed the infection following traumatic brain injury and repeated cerebrospinal fluid shunt procedures. The case highlights an uncommon route of invasive infection in the absence of an underlying immunodeficiency and demonstrates successful management through a multidisciplinary approach involving neurosurgical intervention, molecular identification, antifungal susceptibility-guided therapy, and adjunctive intraventricular liposomal amphotericin B. In addition, we present a comprehensive review of published cases of noncutaneous *Neoscytalidium* infections to place this rare CNS manifestation into its broader clinical and therapeutic context.

### Case presentation

**Fig. 1:**
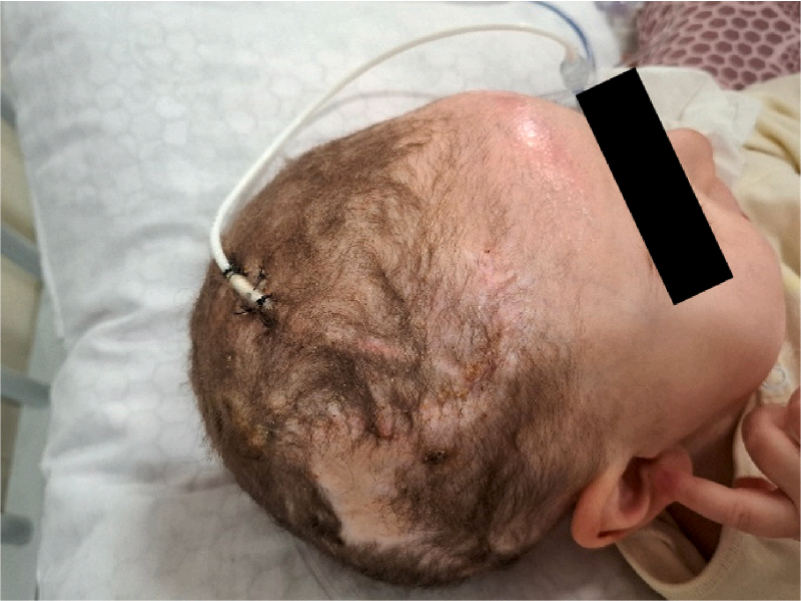
A 1-year-old Iranian boy with a history of road traffic accident and a Cerebral shunt presenting to the Children’s Medical Center in Tehran, Iran.

A 16-month-old Iranian boy with a history of traumatic brain injury following a road traffic accident in October 2024 was admitted on September 13, 2025, with recurrent vomiting and progressive swelling along the ventricular shunt tract. The patient had undergone cerebral shunt placement ten months prior to admission for post-traumatic hydrocephalus and had subsequently required three shunt replacement procedures (Fig. 1).

At presentation, he was referred to the emergency department of Dr. Gharib Children’s Medical Center in Tehran because of persistent vomiting and visible swelling along the shunt tract. Following initial stabilization in the neonatal intensive care unit (NICU), he was transferred to the infectious diseases ward because of clinical suspicion of meningitis. An external ventricular drain (EVD) was inserted to control elevated intracranial pressure and maintain the patient’s level of consciousness.

**Fig. 2:**
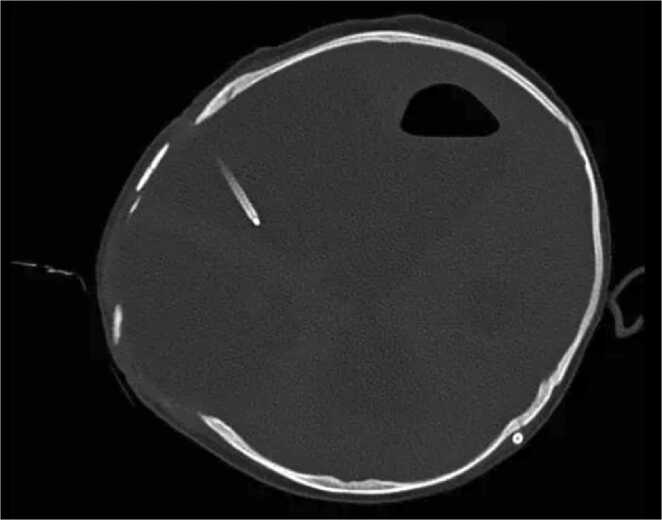
Axial brain CT showing a ventricular shunt, focal skull defect, and a hypodense intracerebral lesion with surrounding edema, consistent with cerebral abscess due to *Neoscytalidium dimidiatum*.

As demonstrated in Fig. 2, axial cranial computed tomography revealed a hypodense intracerebral lesion accompanied by extensive perifocal vasogenic edema and associated mass effect, radiologically consistent with a fungal cerebral abscess. The concomitant presence of a ventricular shunt and focal calvarial defect may represent potential predisposing factors facilitating central nervous system inoculation.

Neuroimaging findings depicted in Fig. 2 were highly suggestive of invasive cerebral mycosis, characterized by a localized hypodense parenchymal lesion with marked surrounding edema, in keeping with previously described radiologic manifestations of fungal brain abscesses caused by dematiaceous fungi. Fig. 2 further illustrates significant perilesional inflammatory changes within the adjacent brain parenchyma, supporting the diagnosis of an aggressive intracranial infectious process attributable to *Neoscytalidium dimidiatum*.

**Fig. 3:**
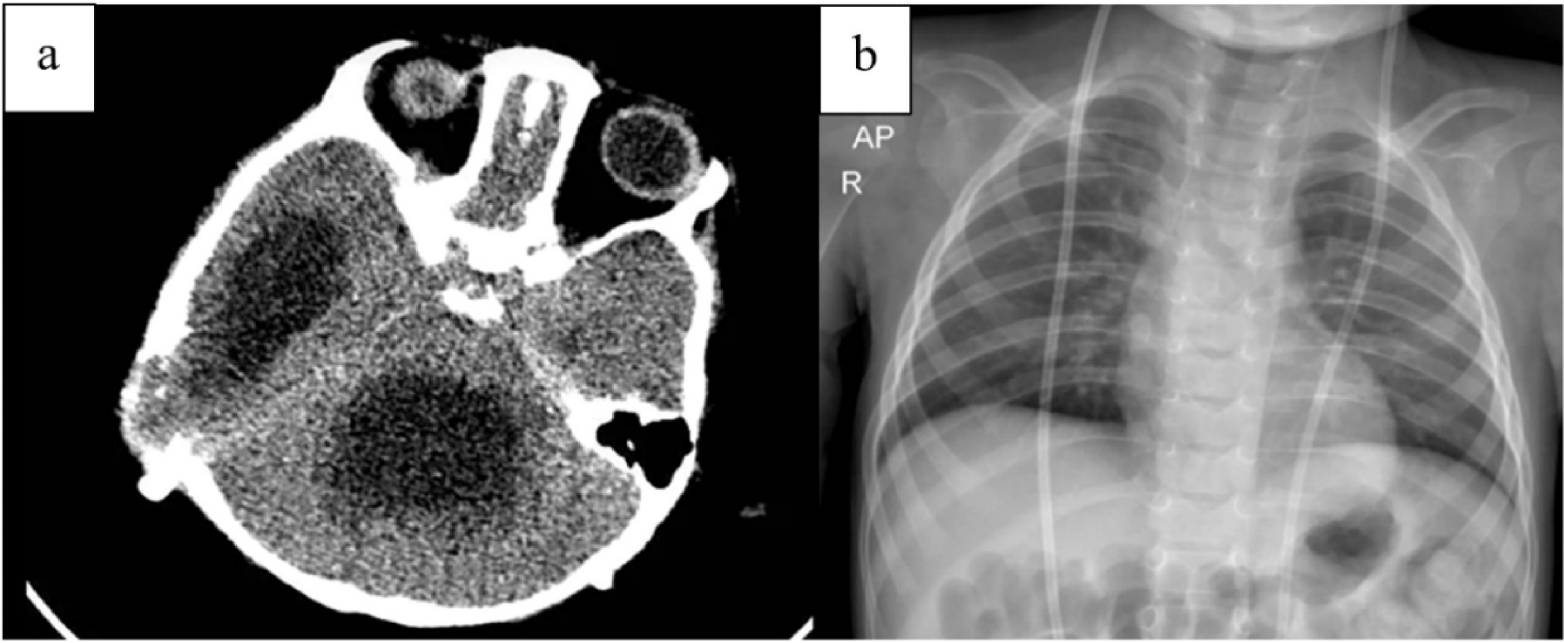
Cranial computed tomography and chest X-ray demonstrating the absence of pulmonary opacities or infiltrative lesions, with no radiologic evidence of pulmonary fungal involvement.

As shown in Fig. 3a, cranial CT imaging confirmed focal intracranial involvement without radiologic evidence of diffuse meningeal extension or multifocal cerebral dissemination. Concurrent chest radiography (Fig. 3b) failed to demonstrate pulmonary infiltrates, nodular opacities, or cavitary lesions suggestive of concomitant thoracic fungal disease. The absence of radiographic pulmonary abnormalities on chest X-ray (Fig. 3b) argues against active pulmonary involvement and supports the likelihood of isolated central nervous system infection. Correlation of neuroradiologic and thoracic imaging findings (Fig. 2, Fig. 3) strongly supports a diagnosis of isolated fungal cerebral abscess in the absence of detectable extracranial dissemination.

Collectively, the imaging findings presented in Fig. 2, Fig. 3 underscore the localized neuroinvasive nature of the infection, with no radiologic evidence of systemic pulmonary spread at the time of evaluation. Radiologic assessment revealed a solitary intracerebral hypodense lesion with surrounding vasogenic edema and preserved thoracic imaging findings (Figs. 2 and 3), a constellation of features compatible with isolated cerebral phaeohyphomycosis.

Based on the initial clinical picture and imaging findings, fungal meningitis, particularly aspergillosis, was suspected. Empirical antifungal therapy with intravenous liposomal amphotericin B (42 mg/day) was initiated. As the patient’s clinical condition deteriorated, the antifungal regimen was modified to a combination of intravenous amphotericin B and caspofungin. On the first day of combination therapy, 30 mg of amphotericin B and 20 mg of caspofungin were administered. From the second day onward, the amphotericin B dose was reduced to 20 mg/day. After six days of combination therapy, amphotericin B was discontinued, and treatment was switched to intravenous fluconazole (6 mg/kg/day, equivalent to 45 mg/day) combined with oral fluconazole (50 mg twice daily). Fluconazole used as a bridging escalation step before the organism was molecularly confirmed. Chosen mainly for its reliable CNS/CSF penetration; its activity against dematiaceous fungi is variable, so it functioned as a holding measure rather than a definitive agent.

Despite this therapeutic intervention, follow-up laboratory evaluations showed no significant clinical or biochemical improvement (Table 1). Hematologic studies revealed anemia, with a hemoglobin level of 9.7 g/dL and hematocrit of 30.3%. The mean corpuscular volume was 70.9 fL and mean corpuscular hemoglobin was 23.1 pg, consistent with microcytic anemia. The total white blood cell count was 6.85 × 10 ³ /μL, with 37% neutrophils, 43.8% lymphocytes, and 17.5% monocytes. Platelet count was elevated at 717 × 10 ³ /μL. Inflammatory markers showed an erythrocyte sedimentation rate of 11 mm/hour and a C-reactive protein level of 4.4 mg/L. HIV serology was negative. Serum immunoglobulin (IgG, IgA, IgM) concentrations were within age-adjusted reference ranges. Peripheral neutrophil count was normal, and no clinical or laboratory evidence of primary or secondary immunodeficiency was identified.

**Table 1:** Summary of Laboratory Diagnostic Findings. Laboratory parameters Measurement Hemoglobin.

**Laboratory parameters**	**Measurement**	**Normal**
**Hemoglobin**	9.7	11–16
**RBC**	4.32	3.5–5.5
**WBC**	6.85	4–10
**HCT**	30.3	37–50
**MCV**	70.9	88–100
**MCH**	23.1	27–31
**Plt**	717	150–450
**ESR**	11	0–10 mm/hour
**CRP (Quantitative)**	4.4	10 >Negative

* RBC, Red blood cell; WBC, White blood cell; MCV, Mean cell volume; MCH, Mean corpuscular hemoglobin; HCT, Hematocrit; ESR, Erythrocyte sedimentation rate; CRP, C-reactive protein.

Cerebrospinal fluid (CSF) analysis demonstrated markedly elevated protein (258 mg/dL) and decreased glucose (37 mg/dL). The CSF appeared semiclear and colorless (Table 2). Arterial blood gas analysis showed a pH of 7.46, PCO₂ of 26.6 mmHg, and bicarbonate level of 18.6 mEq/L, consistent with primary respiratory alkalosis with metabolic compensation (Table 3).

**Table 2:** Cerebrospinal Fluid (CSF) Analysis with focusing on protein, glucose, and CSF characteristics.

Laboratory parameters	Measurement	Normal
Protein (CSF)	258	15–45
Glocos (CSF)	37	60–80 Infant
Color	colorless	-
Appearance	semiclear	-

**Table 3:** Arterial and Venous Blood Gas (ABG/VBG) Parameters.

Laboratory parameters	Measurement	Normal
PH	7.46	7.35–7.45
PCO_2_	26.6	35–45
PO_2_	84	80–100
SO_2_	97.1	95–100
HCO_3_	18.6	22–26
ABE	−3.7	(−2)_(+2)

Because of persistent clinical deterioration, antifungal susceptibility testing (AFST) was performed, and the therapeutic regimen was modified accordingly. AFST was performed using the broth microdilution reference method according to the Clinical and Laboratory Standards Institute (CLSI) document M38, 3rd edition, for filamentous fungi. Amphotericin B, voriconazole, posaconazole, itraconazole, fluconazole, caspofungin, and micafungin were tested. MIC endpoints were determined after 48 h of incubation at 35°C. Since no CLSI or EUCAST clinical breakpoints or epidemiological cutoff values have been established for *Neoscytalidium* species, MIC values were interpreted descriptively and correlated with the patient's clinical response rather than being considered predictive of therapeutic success.

The patient received oral posaconazole syrup (2.5 mL) for two weeks. Toward the end of the second week, intraventricular administration of liposomal amphotericin B via the EVD was initiated at a dose of 0.5 mg, gradually increased to 2 mg over 72 h. After three days of intraventricular amphotericin B, oral posaconazole was continued for an additional month. The patient also received an additional escalating dose of liposomal amphotericin B, which resulted in progressive clinical improvement. Finally, oral itraconazole was administered for two weeks as consolidation therapy. Following the final treatment regimen, both clinical and laboratory parameters showed marked recovery. The patient was discharged one month after stabilization and continued treatment under home-based supervision.

Mycological investigations.

**Fig. 4:**
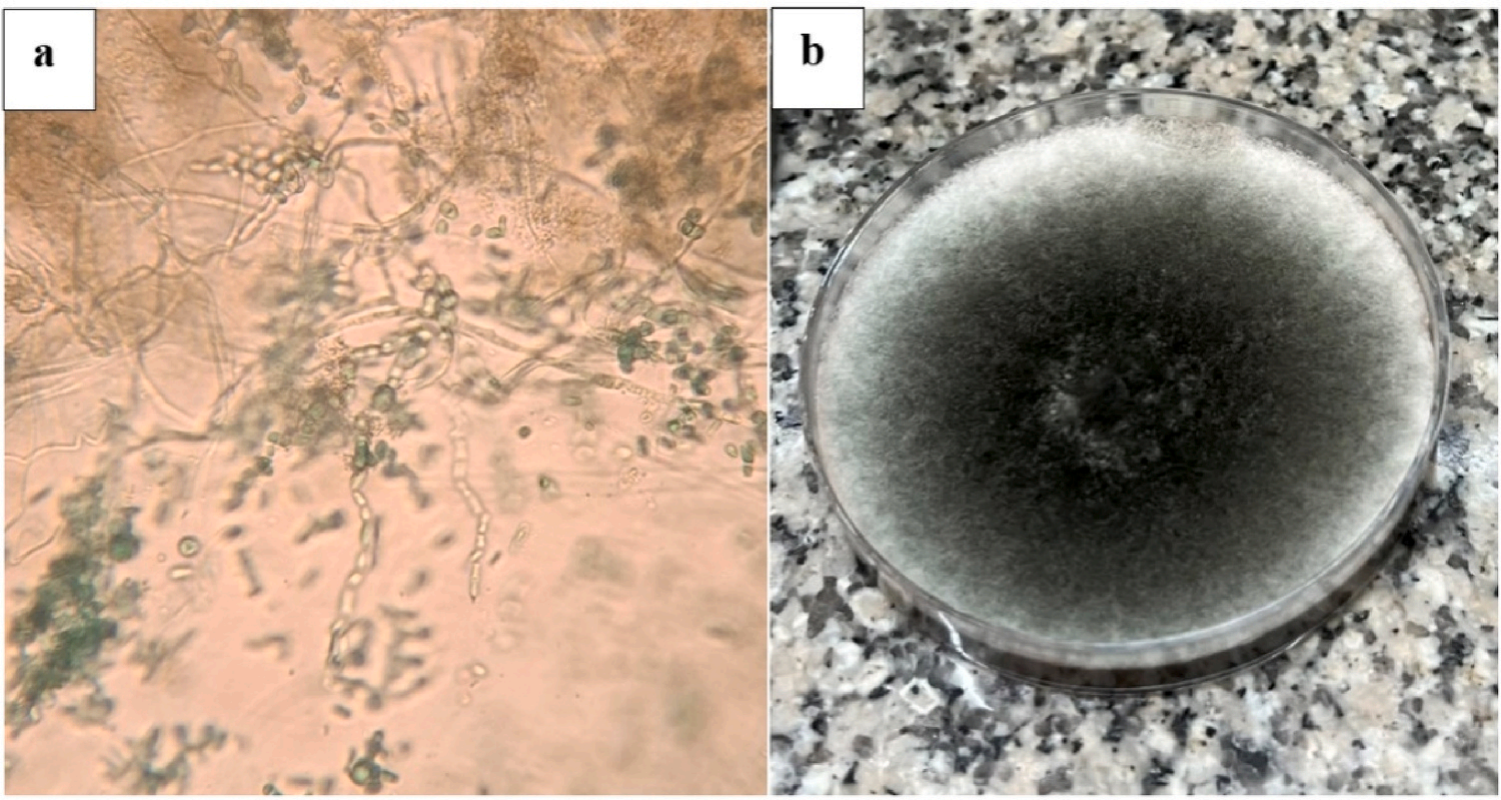
Microscopic features of *Neoscytalidium* and its two-day growth on Sabouraud Dextrose Agar (SDA).

Direct microscopic examination of the CSF revealed dematiaceous, septate hyphae with dark arthroconidia. For culture, CSF samples were inoculated onto Sabouraud dextrose agar and incubated at 30 °C for six days. Initially, colonies appeared white to cream colored; over time, they became olivaceous to dark brown. The reverse side of the colonies showed a dark brown pigmentation, a characteristic feature of *Scytalidium* species (Fig. 4).

Antifungal susceptibility testing was performed using the microdilution method. The minimum inhibitory concentrations (MICs) were as follows: fluconazole, > 64 μg/mL; itraconazole, 4 μg/mL; voriconazole, 2 μg/mL; posaconazole, 2 μg/mL; and amphotericin B, 0.25 μg/mL. Because of the rarity of this pathogen, no established clinical breakpoints are available for interpretation. Nevertheless, amphotericin B demonstrated the lowest MIC among the tested antifungal agents; however, because no clinical breakpoints exist for Neoscytalidium species, MIC values should be interpreted cautiously and cannot independently predict clinical efficacy.

**Fig. 5:**
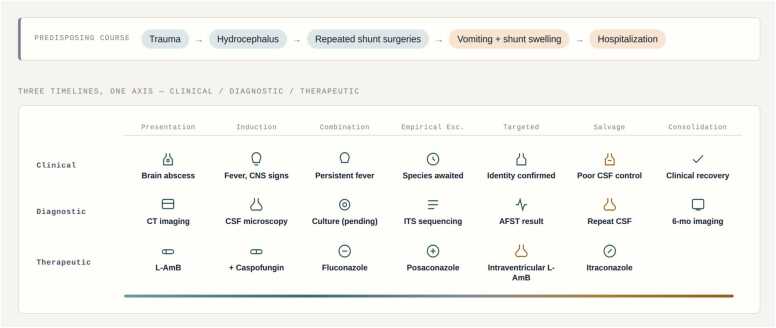
Clinical timeline of the patient's disease course. The timeline summarizes the sequence of major clinical events from traumatic brain injury through neurosurgical interventions, symptom onset, microbiological diagnosis, molecular identification, antifungal susceptibility testing, sequential antifungal therapy, adjunctive intraventricular liposomal amphotericin B administration, clinical recovery, and six-month follow-up without evidence of relapse.

For definitive identification, genomic DNA was extracted from the fungal isolate. Polymerase chain reaction (PCR) amplification of the internal transcribed spacer (ITS) region was performed using the universal primers ITS1 and ITS4. The obtained sequence was analyzed using the Basic Local Alignment Search Tool (BLAST) in the NCBI database, confirming identification as *Neoscytalidium dimidiatum*. The sequence was deposited in GenBank under accession number PX653219 (Fig. 5).

**Fig. 6:**
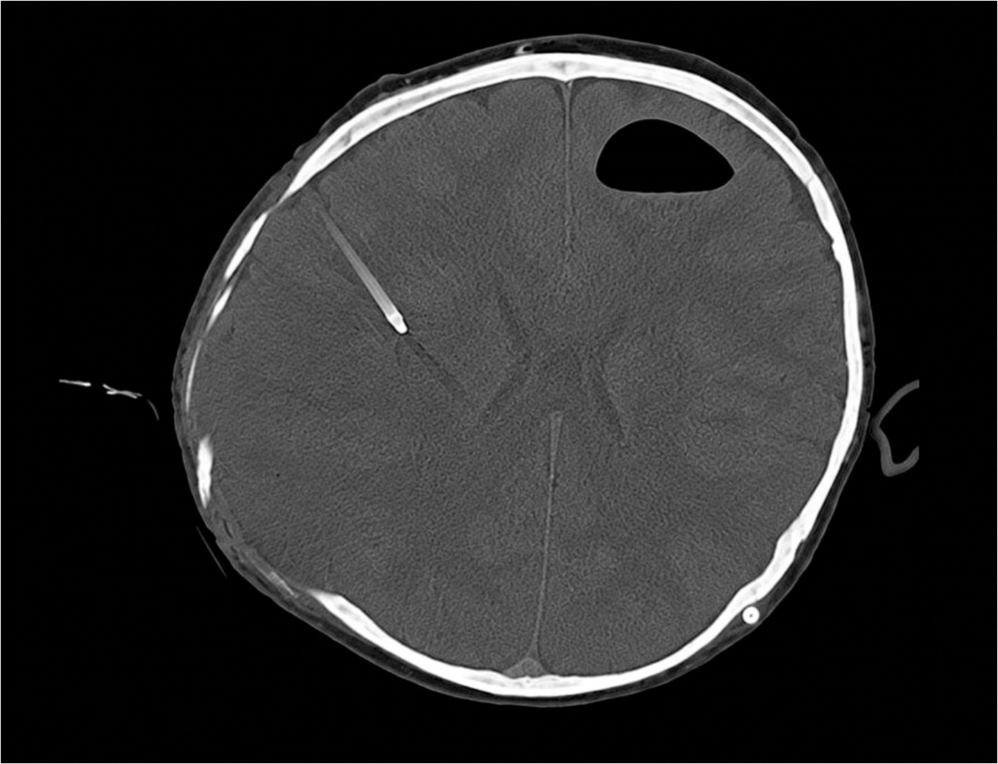
CT images with annotations illustrating the cerebral lesions and their resolution after 6 month treatment.

The patient remained clinically stable during six months of follow-up (to the time of manuscript submission) after completion of antifungal therapy. Serial neuroimaging demonstrated complete resolution of the cerebral lesion, and no clinical or radiological evidence of relapse was observed (Fig. 6).

### Literature search strategy

To provide context for the present case, a structured literature review was performed. Electronic databases including PubMed/MEDLINE, Scopus, Web of Science, and Google Scholar were searched for studies published from database inception to January 31, 2026. The literature search was conducted on February 2, 2026. Search terms included combinations of: “*Neoscytalidium dimidiatum*”, “*Scytalidium dimidiatum*”, “*Nattrassia mangiferae*”, “cerebral phaeohyphomycosis”, “brain abscess”, “central nervous system infection”, “meningitis”, “invasive neoscytalidiosis”, and “systemic Neoscytalidium infection”. Reference lists of all eligible articles were also manually screened to identify additional reports. Publications describing proven or probable noncutaneous *Neoscytalidium* infections in humans were included, whereas duplicate reports, superficial dermatological infections without invasive disease, conference abstracts without sufficient clinical data, and non-English articles lacking adequate clinical information were excluded. Data extracted included patient demographics, immune status, infection site, treatment, surgical intervention, antifungal regimen, and clinical outcome.

## Discussion

Invasive or disseminated infections caused by *Neoscytalidium* species are rare but increasingly recognized (Table 4), and their clinical course is often unpredictable. Reported manifestations include subcutaneous, ocular, sinus, pulmonary, and disseminated infections, occasionally with fatal outcomes, as illustrated by cases such as invasive sinusitis in lung transplant recipients [9]. Although these fungi are primarily known as agents of dermatomycosis and onychomycosis, they have also been implicated in deep-seated and systemic infections.

**Table 4:** Review of studies on reported cases of Subcutaneous and Systemic neoscytalidiosis.*.

**Author/ Year**	**Country**	**Patient**	**Site**	**Species**	**Underlying condition**	**Treatment**	**Outcome**
Mariat/ 1978 [24]	Algeria	30 M	Face, nails	*N. dimidiatum*	Immune deficiency	AmB; griseofulvin	Cured
Dickinson/ 1983 [25]	USA	45 M	Subcutaneous ankle	*S. lignicola*	Diabetes	Debridement + 5-FC	Cured
Zaatari/ 1984 [26]	USA	54 M	Subcutaneous foot	*N. hyalinum*	Immunosuppression	Surgery	Cured
Al-Rajhi/ 1993 [27]	Saudi Arabia	46 M	Endophthalmitis	*N. dimidiatum*	Trauma	Antifungals + surgery	Enucleation
Benne/ 1993 [28]	Netherlands	13 M	Disseminated	*N. dimidiatum*	Lymphoma, neutropenia	AmB	Survived
Levi/ 1994 [29]	USA	32 M	Hand soft tissue	*N. dimidiatum*	Trauma	Surgery + ketoconazole	Chronic morbidity
Rockett/ 1996 [30]	USA	45 F	Subcutaneous foot	*N. dimidiatum*	Transplant, diabetes	Excision + antifungals	Cured
Sigler/ 1997 [7]	USA	73 M	Skin / subcutaneous	*N. dimidiatum*	DM, COPD, CHF	AmB + drainage	Recurrence
Marriott/ 1997 [31]	Australia	44 M	Skin, nodes	*N. dimidiatum*	AIDS	AmB → ketoconazole	Death
Dhindsa/ 1998 [32]	India	24 F	Subcutaneous hand	*N. dimidiatum*	Lupus	Itraconazole	Cured
Gumbo/ 2002 [33]	Zimbabwe	60 M	Endophthalmitis	*N. dimidiatum*	Trauma	Ketoconazole	Improved
Dunn/ 2003 [34]	USA	51 F	Maxillary sinus	*N. dimidiatum*	Lung transplant	Surgery + antifungals	Death
Aamir/ 2003 [35]	Pakistan	20 F	Mycetoma foot	*N. dimidiatum*	Trauma	Ketoconazole	Improved
Morris-Jones/ 2004 [36]	UK	66 M	Cutaneous leg	*N. dimidiatum*	Renal transplant, DM	AmB + terbinafine	Improved
Geramishoar/ 2004 [1]	Iran	17 M	Cerebral abscess	*N. dimidiatum*	SLE, immunosuppression	Surgery + AmB	Death
Willinger/ 2004 [37]	Austria	62 M	Disseminated	*N. dimidiatum*	Renal transplant	AmB → voriconazole	Death
Jabbarvand/ 2004 [38]	Iran	32 M	Keratitis	*N. dimidiatum*	Post-LASIK	PKP	Vision loss
Blázquez/ 2004 [39]	Spain	34 M	Endophthalmitis	*N. dimidiatum*	Trauma	Voriconazole + surgery	Cured
Farjo/ 2006 [40]	USA	21 F	Keratitis	*Scytalidium* sp*.*	Contact lens	AmB + fluconazole	Cured
Pihet/ 2007 [41]	France	58 F	Cutaneous forearm	*N. dimidiatum*	Renal transplant	Excision + itraconazole	Cured
Mani/ 2008 [11]	India	18 M	Cerebral abscess	*N. dimidiatum*	None	AmB + posaconazole	Death
Tan/ 2008 [12]	Canada	31 M	Brain, lung	*N. dimidiatum*	Renal transplant	Itraconazole	Death
Sriaroon/ 2008 [42]	USA	57 M	Subcutaneous leg	*N. hyalinum*	Cardiac transplant, DM	Voriconazole + surgery	Cured
Ikram/ 2008 [43]	Pakistan	19 M	Orbit, sinus	*N. dimidiatum*	None	AmB + itraconazole + surgery	Improved
Elinav/ 2009 [44]	Israel	56 M	Pleural empyema	*N. dimidiatum*	None	Surgery + antifungals	Stabilized
Kindo/ 2010 [45]	India	70 M	Keratitis	*N. dimidiatum*	Trauma	Itraconazole	Unknown
Padhi/ 2010 [46]	India	45 M	Mycetoma foot	*N. dimidiatum*	Trauma	Surgery + antifungal	Improved
Youssef/ 2011 [47]	USA	70 M	Lung nodule	*Scytalidium* sp.	None	Voriconazole	Improved
Moutran/ 2012 [48]	Lebanon	87 M	Cutaneous disseminated	*N. dimidiatum*	Steroids	AmB	Cured
Bakhshizadeh/ 2014 [49]	Iran	20 M	Sinus	*N. dimidiatum*	Asthma, steroids	Surgery	Improved
Hariri/ 2014 [50]	UK	23 M	Pansinusitis	*N. dimidiatum*	Atopy	Surgery + voriconazole	Cured
Sayyad/ 2014 [51]	USA	38 F	Conjunctiva	*Scytalidium* sp.	Contact lens	Natamycin	Cured
Garinet/ 2015 [52]	France	Series	Cutaneous ± disseminated	*N. hyalinum* (1 case) *Neoscytalidium sp.* (1 case)	Variable	Antifungals ± surgery	Mixed
Dionne/ 2015 [53]	USA	50 M	Lung nodules, cavitating	*N. dimidiatum*	Lymphoma (DLBCL)	AmB + Posaconazole	Death
Mishra/ 2015 [54]	India	9 M	Endocarditis	*N. dimidiatum*	None	Surgery + voriconazole	Death
Ho / 2016 [55]	USA	Case Series	Keratitis	*Scytalidium* sp*.* (1 case)	Variable	Surgery + Variable	Mixed
Valenzuela-Lopez/ 2017 [56]	USA	Case Series	35 isolates of 230 Superficial tissue: (66.5% of all) respiratory tract: (17.4%) deep tissues: (9.6%) subcutaneous: (3.9%)	*N. dimidiatum*	NA	NA	NA
Yang/ 2018 [57]	Taiwan	Case Series	Subcutaneous foot	*N. dimidiatum*	Case 1: trauma. Case 2: Diabetes & farming (potential exposure).	Case 1: AmB + Terbinafine Case 2: AmB Voriconazole +	Case 1: Lost to follow-up Case 2: Improved
Abdellaoui/ 2018 [58]	Morocco	62 M	Keratitis secondary Endophthalmitis	*N. hyalinum*	diabetes mellitus	Surgery + Voriconazole	Improved
Calvillo-Medina/ 2019 [59]	Mexico	67 M	Endophthalmitis	*Neoscytalidium oculus*	trauma	AmB + Voriconazole + Natamycin + Surgery	Evisceration
Ghosh /2020 [60]	India	70 M	Keratitis	*Scytalidium lignicola*	trauma	AmB + Itraconazole + Natamycin + Surgery	Perforation Cataract
Alamri/ 2021 [13]	Saudi Arabia	55 F	Cerebral abscess	*N. dimidiatum*	immunosuppression post-renal transplant	AmB + Voriconazole + Surgery	Death
Jo/ 2021 [14]	South Korea	62 M	Cerebral abscess	*N. dimidiatum*	travel to Thailand (endemic region). Liver cirrhosis, HCC surgery, diabetes mellitus	AmB + Voriconazole + Surgery	Cure
Shokoohi/ 2021 [23]	Iran	Cross-sectional	Sinusitis	*N. dimidiatum*	Variable	Surgery + antifungal	Mixed
Heidari/ 2021 [61]	Iran	Retrospective	Respiratory tract	*N. dimidiatum* (n = 8) *Neoscytalidium novaehollandiae* (n = 5)	Variable	Variable	Mixed
Raiesi/ 2022 [62]	Iran	34 F	Sinusitis	*N. dimidiatum*	ABPA؛ nasal polyposis؛ prolonged corticosteroid use	AmB + Voriconazole + Posaconazole+ Surgery	Recovered
Alabdely/ 2023 [15]	Saudi Arabia	Case Series	Cerebral abscess	*N. dimidiatum*	Patient 1: Kidney transplant. Patient 2: Renal transplant.	Patient 1: Surgery + AmB Patient 2: Surgery + AmB + Voriconazole	Both patients died
Ashraf / 2024 [63]	India	57 M	Keratitis	*N. dimidiatum*	trauma	Surgery + Natamycin+ Ketoconazole	Improved
Qi/ 2025 [64]	China	55 F	Endophthalmitis	*N. dimidiatum*	trauma	Voriconazole + Itraconazole+ Surgery	Enucleation
Rivero-Rodríguez et al. (2025) [65]	Spain	76 M	Deep cutaneous	*N. dimidiatum*	Trauma Renal transplant recipient	Terbinafine + Caspofungin+ AmB + Voriconazole + Surgery +	Cured

**Abbreviations**: AmB, amphotericin B; AF, antifungal; CNS, central nervous system; DM, diabetes mellitus; PKP, penetrating keratoplasty; SLE, systemic lupus erythematosus, ABPA: Allergic Bronchopulmonary Aspergillosis.

* Throughout this review, all cases have been uniformly designated by the contemporary nomenclature of the organism *Neoscytalidium* to circumvent confusion resulting from previous taxonomic changes.

**Fig. 7:**
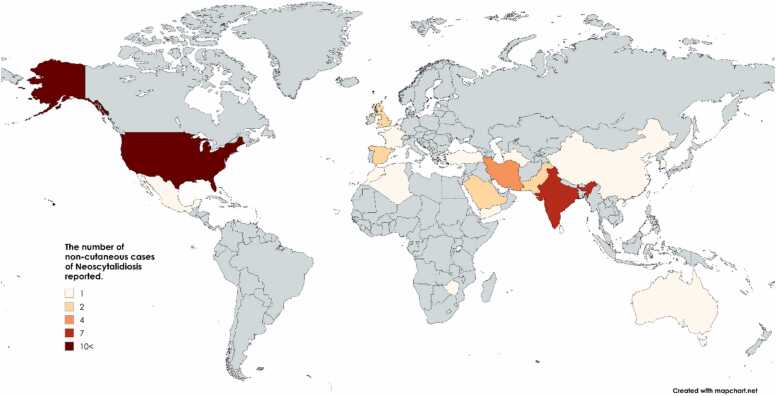
Geographical distribution of the reviewed cases of *Cytalidium* infection. The map was created with mapchart.net.

**Fig. 8:**
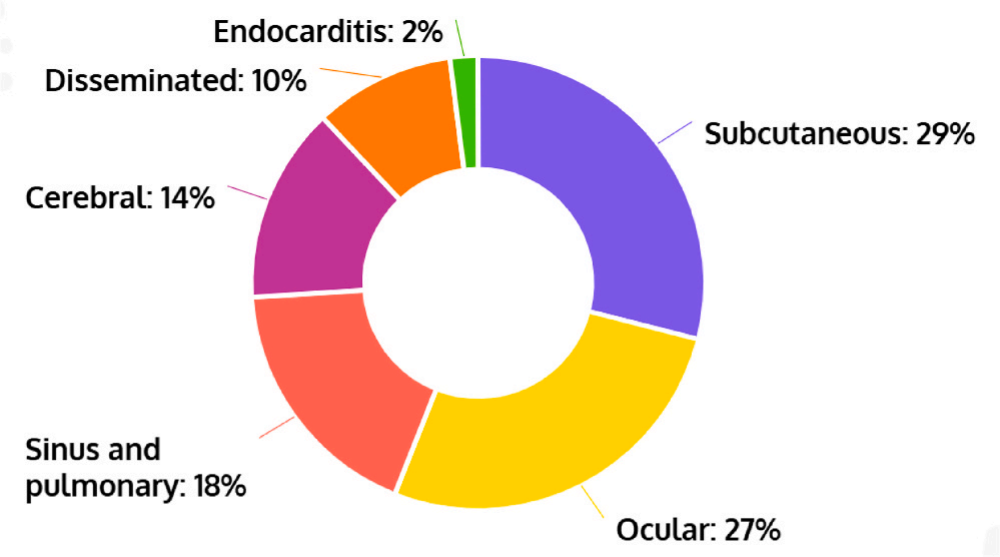
Diversity of non-cutaneous lesions caused by *Neoscytalidium*.spp in 51 patients reviewed in this study (Subcutaneous infection: 15/Ocular infection: 14/Sinus and pulmonary infection: 9/Cerebral infection: 7/Disseminated infection: 5 /Endocarditis: 1).

*Neoscytalidium* species are filamentous fungi endemic to tropical and subtropical regions, including parts of Africa, South America, the Caribbean, India, and Southeast Asia [10]. The epidemiological pattern observed in the cases reviewed in this study demonstrates a clear association between superficial infections and subsequent deep or disseminated disease (Fig. 7). Among reported invasive cases, subcutaneous and ocular infections were the most frequent, accounting for approximately 29.4% and 27.5% of cases, respectively, followed by sinus and pulmonary involvement (17.6%) (Fig. 8).

CNS infection caused by *Neoscytalidium* remains exceptionally rare but carries a very high mortality rate. A review of the literature identified seven reported cases of cerebral infection, six of which resulted in death. In a previously reported case from Iran, a brain abscess caused by cerebral phaeohyphomycosis led to fatal outcome despite treatment with high-dose amphotericin B [1]. Similarly, a case from India described multiple brain abscesses and meningitis caused by *N. dimidiatum*, resulting in death after two months despite treatment with amphotericin B, fluconazole, and subsequently high-dose posaconazole, combined with neurosurgical interventions [11].

Another case from Canada involved a renal transplant recipient who developed concurrent infections with *Macrophomina phaseolina* and *Neoscytalidium dimidiatum*. Despite initial improvement with antifungal therapy, the patient ultimately died from cerebral infection, confirmed by post-mortem CSF culture [12]. Likewise, a kidney transplant recipient in Saudi Arabia developed rapidly progressive CNS disease despite early combination antifungal therapy with liposomal amphotericin B and voriconazole, and died within ten days of diagnosis [13].

In contrast, only one reported case demonstrated successful treatment. This case, from South Korea, involved a patient with liver cirrhosis, diabetes mellitus, and prior hepatocellular carcinoma. Following stereotactic drainage and prolonged amphotericin B therapy, the patient showed gradual radiological and clinical improvement, with complete resolution of lesions after 15 months of follow-up [14]. Additional cases from Saudi Arabia also highlight the poor prognosis of cerebral infections, even with aggressive surgical and antifungal treatment, as both patients ultimately died despite drainage procedures and amphotericin-based regimens [15].

Overall, the limited number of reported cases precludes the establishment of a standardized therapeutic regimen. Among the seven previously reported CNS cases, the mortality rate was approximately 85.7% (six of seven patients). Most affected individuals were severely immunocompromised: four were kidney transplant recipients, one had systemic lupus erythematosus with renal involvement, and another had liver cirrhosis and carcinoma complicated by diabetes mellitus. Only one previously reported patient lacked an identifiable immunodeficiency.

Comparison of the reported CNS cases reveals several clinically relevant prognostic patterns. First, underlying immune status appears to be an important determinant of outcome, as the majority of fatal cases occurred in patients with profound immunosuppression, particularly solid-organ transplant recipients receiving long-term immunosuppressive therapy. Nevertheless, the occurrence of cerebral infection in our immunocompetent patient suggests that disruption of anatomical barriers following traumatic brain injury and repeated neurosurgical procedures may independently facilitate CNS invasion. Second, surgical intervention alone did not consistently improve survival, since several patients died despite abscess drainage or repeated neurosurgical procedures.

In contrast, successful outcomes were observed in patients receiving combined surgical management together with prolonged systemic antifungal therapy. Third, antifungal treatment was highly heterogeneous across reported cases. Although amphotericin B formed the backbone of therapy in most patients, combination regimens incorporating triazoles, particularly voriconazole or posaconazole, appeared more frequently among survivors. Finally, early microbiological diagnosis, molecular confirmation, individualized antifungal therapy guided by susceptibility testing, and multidisciplinary management seem to represent the most important factors associated with favorable clinical outcomes.

The present case broadens the currently recognized clinical spectrum of invasive *Neoscytalidium dimidiatum* infection. Unlike the majority of previously reported cerebral infections, which occurred predominantly in adults with severe underlying immunosuppression, our patient was an immunocompetent infant who developed cerebral phaeohyphomycosis following traumatic brain injury and repeated CSF shunt procedures. This observation suggests that disruption of normal anatomical barriers and the presence of indwelling neurosurgical devices may represent important predisposing factors for CNS invasion even in the absence of classical immunodeficiency. Moreover, successful treatment required repeated neurosurgical management in combination with systemic antifungal therapy guided by antifungal susceptibility testing and adjunctive intraventricular liposomal amphotericin B. The favorable outcome observed in our patient contrasts with the high mortality reported in previously published CNS cases and underscores the potential benefit of early diagnosis, aggressive surgical management, and individualized antifungal therapy.

.Environmental exposure may also play a role in infection risk. *Neoscytalidium* species have been isolated from trees and plant materials in Iran. For example, *Neoscytalidium* was reported from eucalyptus trees in the southern provinces of Ahvaz and Hormozgan [16], and *Neoscytalidium novaehollandiae* has been associated with dieback in *Pinus eldarica* in Tehran and Qazvin provinces [17]. Interestingly, the patient’s accident occurred in Qazvin province, although the infecting species in this case was *N. dimidiatum*.

In addition to human infections, *Neoscytalidium* species have been reported in animals, including a German shepherd dog and a Risso’s dolphin, highlighting the ecological versatility of this genus [18], [19]. Experimental studies in mice have also demonstrated that *N. dimidiatum* exhibits greater pathogenicity than *N. hyalinum*, with a predilection for visceral organs such as the spleen and kidneys [20].

Currently, there is no standardized treatment for systemic *Neoscytalidium* infections. Amphotericin B, voriconazole, posaconazole, and other azoles have been used with variable success [8]. In vitro susceptibility studies of isolates from superficial infections have shown good activity of amphotericin B, miconazole, clotrimazole, and voriconazole, while fluconazole and itraconazole often demonstrate reduced activity or resistance [21]. These findings are consistent with the susceptibility profile observed in the present case, in which amphotericin B demonstrated the lowest MIC among the tested antifungal agents; however, because no clinical breakpoints exist for *Neoscytalidium* species, MIC values should be interpreted cautiously and cannot independently predict clinical efficacy.

Given the high resistance rates and variable susceptibility patterns among *Neoscytalidium* species, accurate and timely identification is essential for appropriate antifungal selection [22]. Molecular methods, particularly ITS sequencing, play a crucial role in confirming the diagnosis, especially in invasive or atypical cases.

A comparison of all published CNS infections caused by *Neoscytalidium dimidiatum* is summarized in Table 5. Despite the limited number of reported cases, several clinically relevant observations can be made. Most previously reported patients had significant predisposing conditions, including solid organ transplantation, hematological malignancies, corticosteroid therapy, or other forms of immunosuppression. In contrast, our patient had no evidence of immunodeficiency and developed cerebral infection following traumatic brain injury and repeated cerebrospinal fluid shunt procedures, suggesting that disruption of normal anatomical barriers and the presence of neurosurgical devices may represent important independent risk factors for CNS invasion. Furthermore, survival appeared to be associated with aggressive neurosurgical management combined with prolonged systemic antifungal therapy, whereas delayed diagnosis and disseminated disease were frequently associated with poor outcomes. Although the small number of reported cases precludes definitive conclusions, these findings highlight the importance of early recognition, prompt surgical intervention when indicated, and individualized antifungal therapy in improving patient outcomes.

**Table 5:** Published cases of central nervous system infections caused by *Neoscytalidium dimidiatum*, including patient characteristics, underlying conditions, antifungal therapy, neurosurgical intervention, and clinical outcome.

Author/Year	Age/ Sex	Immune status	Surgery	AFST-guided	Antifungal	Outcome*
Geramishoar et al. / 2004 [1]	17 years /male	Immunocompromised (SLE, on prednisolone & cyclophosphamide)	Yes	No	Amphotericin B	Died (within 1 week)
Mani et al. / 2008 [11]	18 years /male	Immunocompetent	Yes	Yes	Amphotericin B + Fluconazole, then switched to Posaconazole	Died (after 8 weeks of hospitalization)
Tan et al. / 2008 [12]	31 years /male	Immunocompromised (due to living-related renal transplant; on prednisone, mycophenolic acid, cyclosporine, tacrolimus, and thymoglobulin for acute rejection)	Yes	Yes	Voriconazole (initial response), then itraconazole + empiric amphotericin B (during final deterioration)	Died (after acute neurological deterioration; CSF grew *S. dimidiatum* post-mortem)
Alamri et al. / 2021 [13]	55 years /female	Immunocompromised (due to living-related renal transplant; received anti-thymocyte globulin [ATG] for induction, maintained on prednisolone, tacrolimus, and mycophenolate-mofetil)	Yes	No	Liposomal amphotericin B + voriconazole	Died (on Day +47 post-transplant)
Jo et al. / 2021 [14]	62 years (male, Korean)	Immunocompromised (underlying liver cirrhosis, hepatocellular carcinoma [HCC], and diabetes mellitus; no transplant)	Yes	No	Amphotericin B (prolonged course; total ∼16 weeks). Voriconazole was tried but ineffective.	**Alive / Cured** (lesions significantly improved on MRI, dysarthria and weakness resolved, followed for ∼15 months)
Alabdely et al. / 2023 [15]	36 years (male)	Immunocompromised (due to renal transplant)	Yes	No	Amphotericin B	Died
	55 years (female)	Immunocompromised (due to renal transplant)	Yes	No	Liposomal amphotericin B + Voriconazole	Died
Current case/2026	16 month	Immunocompetent	Yes	Yes	VP shunt replacement + systemic voriconazole + intraventricular amphotericin B	Alive

* Mortality: 5/7 previous reports

Although amphotericin B was included in the treatment regimen of most reported CNS cases, the antifungal strategies varied considerably, reflecting the absence of standardized therapeutic recommendations for cerebral *Neoscytalidium* infection. Likewise, patient outcomes were heterogeneous and appeared to depend not only on the antifungal regimen but also on host immune status, timing of diagnosis, extent of CNS involvement, and the feasibility of surgical source control. In our patient, repeated neurosurgical interventions, antifungal susceptibility-guided modification of therapy, and adjunctive intraventricular liposomal amphotericin B were considered complementary factors that likely contributed to the favorable clinical outcome.

## Conclusion

Cerebral phaeohyphomycosis caused by *Neoscytalidium dimidiatum* remains an exceptionally rare but potentially fatal infection. This case expands current knowledge by demonstrating that invasive CNS infection may occur in immunocompetent infants following traumatic brain injury and repeated CSF shunt procedures. Early molecular identification, careful interpretation of antifungal susceptibility results, and close collaboration between neurosurgeons, microbiologists, and infectious diseases specialists were essential for successful management. Continued accumulation of well-documented cases will be important to better define optimal therapeutic strategies and prognostic factors for this uncommon but life-threatening infection.

To the best of our knowledge, and based on our comprehensive review of the published literature, this is the first reported pediatric case of CNS infection caused by *Neoscytalidium dimidiatum* that resulted in complete recovery. This case therefore expands the currently recognized clinical spectrum of invasive *N. dimidiatum* infection and demonstrates that favorable outcomes can be achieved through timely diagnosis, repeated neurosurgical source control, and individualized antifungal therapy.

## CRediT authorship contribution statement

**Hamid Eshaghi:** Supervision, Resources, Formal analysis, Data curation, Conceptualization. **Mahmoud Khansari:** Visualization, Validation, Software, Methodology, Investigation. **Shahram Mahmoudi:** Writing – original draft, Software, Resources, Investigation. **Kimia Kamali Sarvestani:** Software, Formal analysis, Data curation. **Hasti Kamali Sarvestani:** Supervision, Project administration, Methodology, Conceptualization. **Fuad Haghighat:** Writing – review & editing, Writing – original draft, Visualization, Resources, Investigation, Formal analysis.

## Ethical considerations

The data and findings presented in this article are original and have not been published elsewhere. Each author has contributed meaningfully to this study and endorses the final version of the manuscript.

## Funding

This study was conducted independently, without receiving financial support from any public, commercial, or not-for-profit funding agencies. The research, from its conception to publication, was solely the responsibility of the authors.

## Declaration of Competing Interest

The authors declare no competing interests.
